# Alphavirus Virulence Determinants

**DOI:** 10.3390/pathogens10080981

**Published:** 2021-08-03

**Authors:** Margarita V. Rangel, Kenneth A. Stapleford

**Affiliations:** Department of Microbiology, New York University Grossman School of Medicine, New York, NY 10016, USA; Margarita.Rangel@nyulangone.org

**Keywords:** alphavirus, virulence factors, pathogenesis, transmission

## Abstract

Alphaviruses are important pathogens that continue to cause outbreaks of disease in humans and animals worldwide. Diseases caused by alphavirus infections include acute symptoms of fever, rash, and nausea as well as chronic arthritis and severe-to-fatal conditions including myocarditis and encephalitis. Despite their prevalence and the significant public health threat they pose, there are currently no effective antiviral treatments or vaccines against alphaviruses. Various genetic determinants of alphavirus virulence, including genomic RNA elements and specific protein residues and domains, have been described by researchers to play key roles in the development of disease, the immune response to infection, and virus transmissibility. Here, we focus on the determinants that are currently described in the literature. Understanding how these molecular determinants shape viral infections can lead to new strategies for the development of therapies and vaccines to combat these viruses.

## 1. Introduction

Alphaviruses are enveloped single-stranded positive-sense RNA viruses that belong to the family *Togaviridae*. Many members of the alphavirus genus cause disease in humans and animals and are transmitted by several widespread mosquito species [1,2,3]. The wide distribution of competent vectors, increasing global transportation, disturbance of landscapes, and climate change, among other factors, continue to cause mosquito-borne diseases to emerge and re-emerge [4,5,6]. This is demonstrated by chikungunya virus (CHIKV), a prevalent alphavirus, that for decades following its initial detection, did not cause large outbreaks and was limited to parts of Africa and Asia. It was not until the early 2000s that explosive outbreaks occurred and led to spread of CHIKV to new regions in Europe and the Americas [7,8]. The Old World alphaviruses, which include CHIKV, Semliki Forest virus (SFV), Sindbis virus (SINV), O’nyong-nyong virus (ONNV), and Ross River virus (RRV), cause mostly arthritic disease while the New World alphaviruses, which include Mayaro virus, Eastern, Western, and Venezuelan equine encephalitis virus (EEEV, WEEV, and VEEV), are mostly encephalitogenic [9,10]. The alphaviruses can each be further divided into distinct phylogenetically divergent lineages and sublineages. Importantly, lineage-dependent differences in virulence have been observed, indicating virulence is modulated by specific genome-encoded determinants [11].

**Figure 1:**
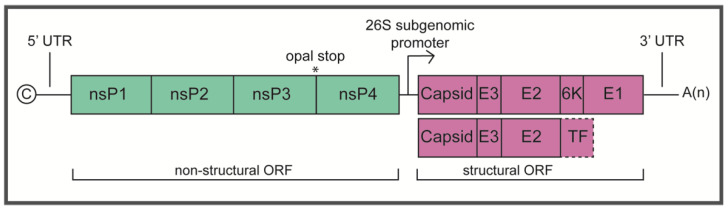
Schematic representation of the alphavirus genome.

**Figure 2:**
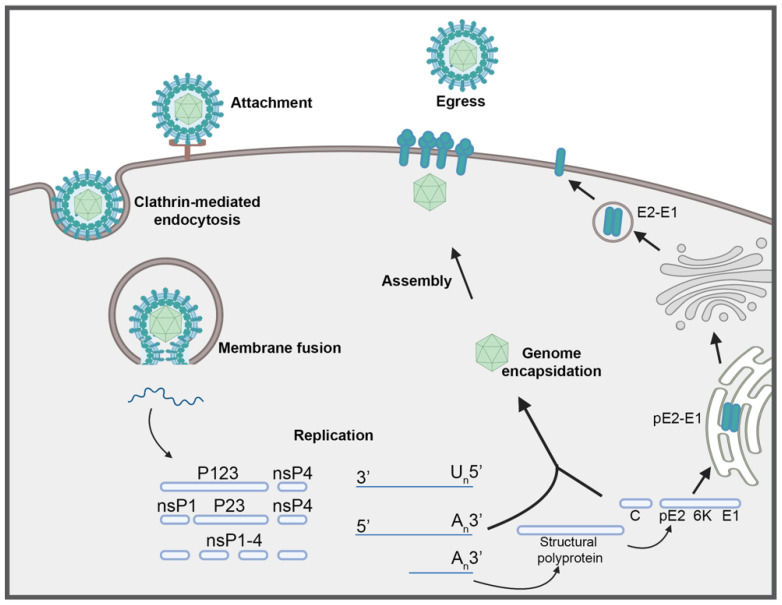
Schematic representation of the alphavirus lifecycle.

Alphaviruses are structurally similar and share a common lifecycle. Mature particles consist of a nucleocapsid core surrounded by a host-derived lipid bilayer studded with transmembrane glycoproteins, E1 and E2, which are arranged in trimeric spikes of heterodimers on the surface of the particle [12]. Alphaviruses have been shown to engage various cell surface attachment factors and putative receptors that facilitate the early stage of cell entry, followed by clatherin-mediated endocytosis and membrane fusion within the early endosome [13,14,15]. Following the delivery of the genome into the cell, viral replication occurs in membrane-associated spherules on the plasma membrane [16,17,18]. The genome is about 12 kb in length, encodes 2 open reading frames (ORFs) flanked by 5′ and 3′ untranslated regions (UTRs) and contains a 5′ methylguanylate cap and 3′ polyadenylated tail (Figure 1). The first ORF encodes a polyprotein that is cleaved into four non-structural proteins (nsP1–4), with cleavage intermediates that function in RNA replication (Figure 2). The second ORF is translated from a subgenomic RNA and encodes the structural polyprotein that is cleaved into 5 structural proteins (capsid, E3, E2, 6K, and E1) or alternatively, a frameshifting event leads to a truncated polyprotein to produce capsid, E3, E2, 6K, and TF. During replication, the non-structural polyprotein P1234 is initially cleaved in *cis* into P123 and nsP4, which together form an unstable early replication complex to synthesize negative-strand RNA. P123 is then cleaved in trans to produce nsP1, P23, and nsP4 that form a replication complex for genomic RNA synthesis. Cleavage into all four non-structural monomers then shifts replication activity to genomic and sub-genomic RNA synthesis [19,20]. Importantly, while the non-structural polyprotein and the cleavage intermediates function in genome replication, the individual proteins are widely multifunctional in subsequent steps of alphavirus infection and contribute largely to virulence in the host. Following translation of the subgenome, the structural polyprotein undergoes processing required for assembly and budding. Viral encapsidation and budding involve nucleocapsid formation in the cytoplasm, processing, and transport of the glycoproteins to the cell membrane, and egress at the cell membrane where the nucleocapsid is enveloped in the membrane-deposited glycoproteins [21,22,23] (Figure 2).

Each component of the genome and the encoded proteins plays a multifunctional role in the alphavirus lifecycle. Through mutational studies and genome mapping, researchers have begun to expand our understanding of these functions to how viral components are able to not only contribute to establishing infection, but also modulate virulence [24,25]. Understanding these determinants and identifying the common mechanisms of virulence can be instrumental in developing tools to prevent the spread of alphaviruses, such as attenuated strains in the development of vaccines. Therefore, we will review here the reported genetic determinants of alphavirus virulence, with a focus on non-synonymous changes, organized by region of the genome that have been shown to impact the development of disease, the immune response to infection, or virus transmissibility as supported by in vivo and/or in vitro experiments. A summary of determinants can be found in Table 1.

## 2. Determinants of Virulence

### 2.1. Non-Structural Proteins

#### 2.1.1. nsP1

Alphavirus nsP1 primarily functions in genome replication in complex with the other non-structural proteins. In particular, the alphavirus capping mechanism requires nsP1, which possesses methyltransferase and guanylylation activities that catalyze steps preceding in the eventual addition of m^7^GMP onto the 5′end of viral RNA [78]. In addition to these roles in replication, nsP1 has also be found to contribute to virulence. A comparison of non-neurovirulent versus neurovirulent SINV strains revealed one single non-synonymous change in nsP1, I538T, which when introduced to a non-neurovirulent strain enhanced neurovirulence in mice, without having a significant impact on replication [26]. Residue 538, located at the conserved nsP1/2 cleavage site, was found to be involved in regulating type I IFN (IFN-I), in a manner independent of host shut off (transcription/translation) and this was observed in both SINV and RRV [27]. nsP1 was also found to be critical in musculoskeletal inflammation during RRV infection independent of viral load, as demonstrated by genome mapping using chimeric viruses [79]. A subsequent study confirmed six amino acids (S79C, A112S, L224I, C416F, S424N, and L463I) that differed between RRV strains used in constructing the chimeric viruses to confer the observed phenotype and attributed the mutations to tissue type-specific IFN-I sensitivity [28]. While single mutants were not sufficient for the phenotype, a double mutant sustaining S79C and L224I sufficiently exhibited an attenuated phenotype in vivo [28]. A recent study further demonstrated a role for nsP1 in regulating the IFN-I response by identifying the CHIKV-mediated degradation of the cytosolic DNA sensor cyclic GMP-AMP synthase (cGAS) and a direct interaction of nsP1 with stimulator of interferon genes (STING) that stabilizes nsP1 and increases palmitoylated nsP1 in vitro, a post-translational modification previously implicated as important in replication and pathogenesis in vivo [80,81,82]. Finally, key residues located at the SFV nsP1 P1/2 cleavage site have been shown to function together with residue 515 of nsP2 and drive neurovirulence in mice [83]. In addition to its functions in viral genome replication, nsP1 is multifunctional with a significant role in controlling the host IFN-I response. Further studies of nsP1-host protein interactions and the function of post-translationally modified nsP1 will provide a better understanding of how nsP1 functions as a virulence determinant to drive alphavirus infections.

#### 2.1.2. nsP2

**Figure 3:**
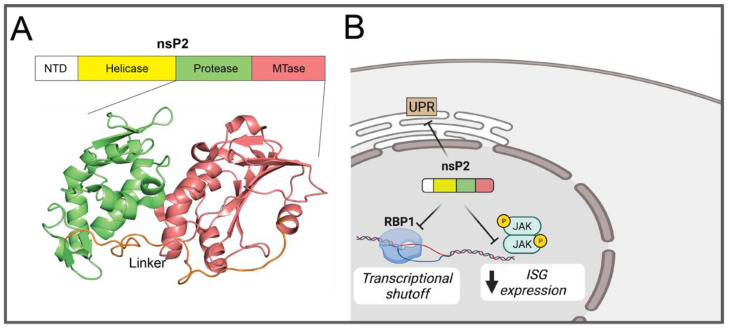
(**A**) Top: Schematic of nsP2 domains. NTD; N-terminal domain. Bottom: Crystal structure of CHIKV nsP2 protease (PDB 3TRK) with the N-terminal protease subdomain (green), C-terminal MTase subdomain (red), and subdomain linker (orange) indicated (**B**) Schematic representation of nsP2-mediated antagonism of host cell processes.

During the alphavirus lifecycle, nsP2 functions as a protease in the processing of the non-structural polyprotein, as a helicase during replication, and exhibits 5′ triphosphatase activity during capping of viral RNAs [84,85,86]. Structurally, nsP2 consists of an N-terminal domain (NTD), a helicase domain containing 5′ triphosphatase activity, a papain-like cysteine protease subdomain, and a C-terminal S-adenosyl-L-methionine (SAM)-dependent RNA methyltransferase-like (SAM MTase-like) subdomain, connected by a ~30 amino acid random coiled linker (Figure 3A) [87,88,89]. As a determinant of virulence, nsP2 can translocate to the nucleus and cause the degradation of a catalytic subunit of DNA-dependent RNA polymerase II (RPB1), resulting in host transcription shut off and inhibiting the activation of anti-viral responses [29,90,91]. This is unique to Old World alphaviruses while similar functions are carried out by the capsid protein of New World alphaviruses (discussed below) [88]. The single point mutation P726G in SINV nsP2 leads to decreased replication and cytopathogenicity in mammalian cells, increased levels of IFN in vitro and in vivo, and an attenuation of the virus in mice [29]. Further mutational analysis of residue 726 demonstrated its modulation of SINV pathogenicity in mice and JAK-STAT inhibition [30,31]. A similar reduction in JAK-STAT inhibition, independent of host shut off, was seen in CHIKV with an nsP2 mutation from a conserved proline to serine at position 718 and the authors propose a decrease in blocking of STAT1 nuclear localization may explain increased IFN levels in nsP2 mutants [31]. In a comparison of field strains of RRV that differ in disease severity in mice, naturally occurring determinants of IFN-I modulation were identified in nsP2 [32]. nsP2 mutations A31T, N219T, S580L, and Q619R each led to higher induction of IFN-I, along with increased RIG-I and/or IRF3 expression [32]. Notably, residue 619 is near a recently identified highly variable loop (VLoop) on the surface of nsP2 SAM MTase-like domain found to be critical for the transcription inhibitory effects in SINV without altering replication [89]. Mutations introduced to nsP2 residues between positions 674 and 688 prevented RPB1 degradation and transcription shutoff and increased IFN induction [89]. A similar peptide was identified in CHIKV (_674_ATLG_677_) that when mutated decreased the transcription inhibitory function of nsP2 without altering replication in murine cells [33]. nsP2 is also able to inhibit the unfolded protein response (UPR) of host cells as a result of transcriptional shutoff and mutations KR649AA and P718S in SINV disrupts this function [34]. Together, these studies demonstrate that nsP2 is a critical, multifunctional determinant of alphavirus virulence with functions including the degradation of RBP1, leading to host transcriptional shutoff and suppression of the UPR, and JAK-STAT inhibition that limits ISG expression (Figure 3B). While the activities of both nsP1 and nsP2 result in modulation of the IFN-I response, it is remarkable to note the evolution of differing mechanisms that provide a multi-pronged strategy of controlling the host. Blocking of the UPR as a result of host transcriptional shutoff by nsP2, for instance, suggests the potential for anti-alphavirus activity by an activated UPR during infection, emphasizing a possible therapeutic target.

#### 2.1.3. nsP3

**Figure 4:**
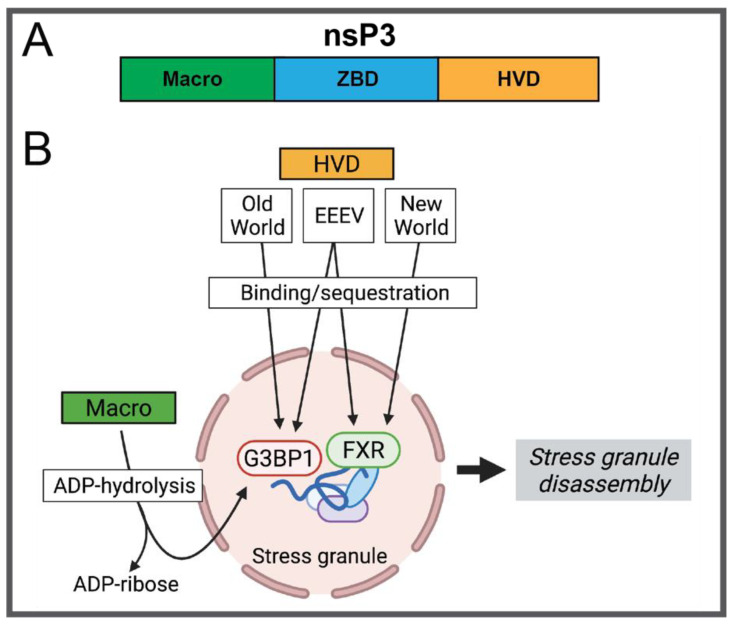
(**A**) Structural arrangement of alphavirus nsP3 subdomains (**B**) Schematic representation of nsP3-mediated stress granule disassembly.

Alphavirus nsP3 functions as part of the replication complex, induces plasma membrane remodeling in the formation of spherules where replication takes place, and is the only non-structural protein that is phosphorylated, which is important for efficient RNA synthesis [35,92,93]. nsP3 exhibits multiple functions through the alphavirus lifecycle with significant roles in virulence in addition to replication. The protein consists of a conserved N-terminal macrodomain and a C-terminal intrinsically disordered hypervariable domain (HVD), joined by a central zinc-binding domain (ZBD) (Figure 4A) [90,91]. The nsP3 macrodomain possesses ADP-ribosyl-binding and hydrolase activity that modulates neurovirulence, as demonstrated by mutations of SINV nsP3 residues 32 and 114 altering pathogenesis in mice and macrodomain activity in vitro [39]. Importantly, the macrodomain ADP-ribosylhydrolase is crucial for the suppression of stress granule formation late in viral infection by targeting Ras GTP-activating protein-binding protein G3BP1, that functions in stress granule assembly (Figure 4B) [94]. The C-terminal HVD harbors multiple host protein interacting sites, including binding motifs for G3BPs. The function of nsP3 in controlling the cellular stress response by causing a disassembly of stress granules demonstrates the elaborate multifunctionality of the tripartite protein. Following binding of G3BP1 and/or FXR by the HVD, the macrodomain ADP-hydrolase activity removes ADP-ribose from the stress granule associated proteins, leading to granule disassembly (Figure 4B). The HVD of the highly pathogenic New World alphavirus EEEV nsP3 was shown to be critical for neurovirulence as deletion of G3BP-binding motif (nsP3 471–483) and/or its FXR-binding motif (nsP3 531–547) decreased neurovirulence in mice [40]. The HVD of EEEV nsP3 possessing both FXR and G3BP binding motifs is unique as other New World alphaviruses interact with members of the FXR family, but not G3BPs, and Old World alphavirus HVDs interact with G3BPs, but not FXR family members. This observation is monumental in understanding how EEEV is able to, in part, achieve its extremely high virulence by exhibiting a wider range of host protein binding partners. Importantly, while these HVD-host protein interactions are critical for virulence, they also drive efficient viral replication, presumably making the redundancy of binding both G3BPs and FXR family members beneficial to viral fitness. Additional interactions between nsP3 HVDs of both Old and New world alphaviruses and cytoskeletal proteins, have been identified and demonstrated to be important for infectivity [95].

Additionally, the nsP3 HVD also contains multiple phosphorylation sites critical for replication and virulence. Phosphorylation sites, T344 and T345, modulate SFV neurovirulence in mice [35] and SINV neurovirulence was attributed to an 18-amino acid deletion from nsP3 residue 386 to 403, without impacting replication in cell culture, that resulted in the removal of seven serine residues, suggested to effect the overall phosphorylation of nsP3 [36]. Further, multiple alphavirus genomes contain an opal termination codon at the end of the nsP3 gene, which is read through for the translation of nsP4 and therefore modulates the production of the alphavirus RNA-dependent RNA polymerase. Importantly, altering of this codon modulates neurovirulence in SINV (Opal-537) and SFV (Opal-469) and arthritis induced by CHIKV (Opal-524) [36,37,38]. These findings indicate that in addition to roles in achieving optimal viral replication, the diverse and multifunctional domains of nsP3 carry out a wide range of activities that drive alphavirus virulence.

#### 2.1.4. nsP4

Alphavirus nsp4 is the core RNA-dependent RNA polymerase (RdRp), which together with polyprotein nsP123 synthesizes minus strand RNA and then forms the late replication complex with fully processed nsP1–3 to produce genomic and subgenomic plus strand RNA [19]. Additionally, nsP4 possesses adenylyltransferase activity that may function in the addition of the 3′ poly(A) tail [19,92]. RdRp fidelity in many viruses, including alphaviruses, has been recognized as a contributor to viral fitness and pathogenicity in animals [41,93]. The high fidelity CHIKV nsP4 mutant C483Y yielded lower infection and dissemination in mosquitoes and lower titers in mice, which, as discussed by the authors, is likely attributed to decreased population diversity [41,96]. This was also observed in VEEV, as RdRp mutations G7R, E31G, S90T, and C482Y led to an increased sensitivity to bottlenecks in mice and mosquitoes and an altered viral diversity [42]. Alphavirus fidelity variants are now being used as candidate vaccine platforms for CHIKV and other alphaviruses [97,98,99]. Finally, although specific functional residues have not been identified, subcloned nsP4 of CHIKV and SINV transfected into human cells blocks the phosphorylation of eIF2α, a key player in the UPR of host cells, indicating the nsP4 gene encodes a determinant able to counteract this measure of host defense [43]. This demonstrates yet another complementary virus-induced blocking of the UPR, indicating a potentially potent antiviral activity that could be harnessed by a UPR-focused therapeutic approach. Further studies of nsP4 will be incredibly insightful into alphavirus replication and antagonism of host processes.

### 2.2. Structural Proteins

#### 2.2.1. Capsid

The alphavirus capsid protein functions in genome encapsulation, particle assembly, and budding [100]. To achieve these functions, it contains regions that interact with viral genomic RNA and the viral glycoproteins [100,101,102]. In New World alphaviruses, capsid is an important virulence determinant involved in innate immune response suppression through host transcriptional shut off, while in Old World alphaviruses similar processes are mediated by nsP2 [88]. An N-terminal peptide of VEEV capsid was found to accumulate at the nuclear membrane and cause cytopathogenicity to the extent of full-length capsid and it was suggested that this region of the protein contains a nuclear localization signal (NLS) enabling it to block nuclearcytoplasmic transport in infected cells [44]. Further, a nuclear export signal (NES) was identified in VEEV capsid and mutations L48A and F50A abolished inhibition of nuclear import by the N-terminal peptide [45]. In EEEV, a 20-amino acid region of capsid (55–75) was also linked to inhibition of host gene expression and sensitivity to interferon and within this region residues 65–69 were identified as putative NLS [46]. Both Δ55–75 and Δ65–69 EEEV mutants exhibit decreased virulence in mice [46]. While the capsid of WEEV, which has a nucleocapsid arrangement more similar to Old World alphaviruses, has not been linked to transcriptional shut off, it has been shown to antagonize pattern recognition receptor (PRR) pathways downstream of interferon regulatory factor 3 (IRF-3) [47,48]. CHIKV capsid was also found to contain an NES and interact with nuclear export protein CRM-1, but blocking of this interaction was not as detrimental as seen in the New World alphaviruses [103]. Additional roles of capsid in virulence have been characterized in the Old World alphaviruses. In a study that sought to identify interactions between SINV capsid and viral cytoplasmic RNA identified discrete interacting regions of the genome, using CLIP-seq analysis, that when mutated resulted in greater interferon production in cell culture and attenuation in mice [104]. The authors propose that following entry into host cells and nucleocapsid assembly, continued capsid-genome interactions enable efficient genomic RNA function, contributing to the efficient establishment of infection and modulation of the innate immune response and consequent pathogenesis [104]. Recently, SFV capsid was found to interfere with RNAi in mammalian cells through sequestration of double-stranded RNA and small interfering RNA and this activity was abrogated in K124A/K128A and K139A/K142A capsid mutants [49]. Such findings demonstrate the wide variety of intracellular interactions mediated by the alphavirus capsid protein. Harboring diverse interactions with other structural proteins and genomic RNA and contributing to host shutoff and antagonism of the host immune response, the capsid protein further emphasizes the multifaceted strategy employed by alphaviruses in determining virulence throughout the viral lifecycle that incorporates both non-structural and structural proteins.

#### 2.2.2. E1 and E2 Glycoproteins

**Figure 5:**
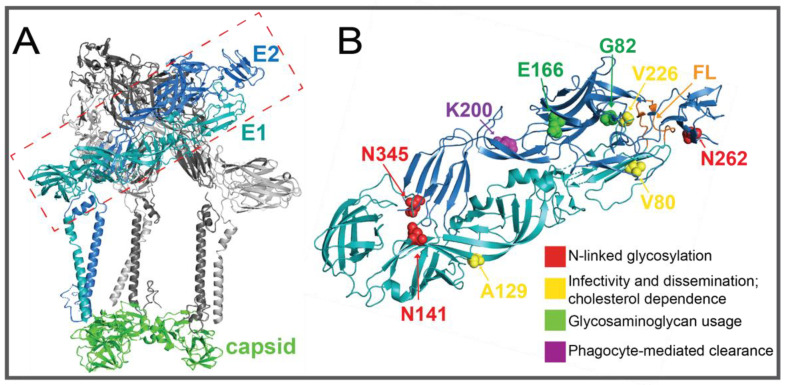
(**A**) CHIKV glycoprotein spike (PDB 3J2W; [116]) (**B**) CHIKV E1/E2 heterodimer with determinants of virulence indicated (PDB 2FXB; [117]).

The surface of alphavirus particles contains 240 copies of each transmembrane glycoprotein, E1 and E2, which are about 50 kDa in size and primarily composed of beta-sheets (Figure 5A) [105]. E2 is recognized as the attachment protein, which binds host cell receptors and entry factors, and E1 as a class II fusion protein that mediates membrane fusion. They exhibit dimeric interactions that are important in protein processing, trafficking to the membrane, and particle assembly, in addition to their primary roles in attachment and fusion. Early studies utilizing chimeric viruses with swapped domains of virulent and avirulent strains of alphaviruses began to highlight discrete regions of E1 and E2 as important for virulence. In one such study, chimeric viruses were constructed to dissect the varying neurovirulence between different strains of neuro-adapted SINV in adult and infant wild-type mice [50]. The mutations V72A and G313D in E1 and G55H and L209G in E2 were linked to increased neurovirulence [50]. G55H and L209G in E2 were attributed to increased neurovirulence in both adult and infant mice [50]. Subsequent mechanistic studies demonstrated G55H to increase infectivity in neural cells and to also require a second mutation, E70K, to confer full virulence that the researchers attributed to increased binding to the glycosaminoglycan (GAG) heparan sulfate (HS), a ubiquitously expressed host factor used by viruses and other pathogens for cell attachment [106,107]. Similarly, other groups used this domain-switching approach in SFV to identify E2 as containing determinants of virulence [108]. Increased GAG dependence of neuroinvasive SFV was correlated to more efficient crossing of the blood–brain barrier [109]. There has been continued evidence that GAG usage contributes to alphavirus virulence and that binding is mediated by specific regions of E2, such as residues 70, 82, and 166 of CHIKV E2 that were identified using the attenuated strain 181/25 and were shown to modulate disease severity and host response (Figure 5B) [58,59,60]. Enhanced neurovirulence in EEEV is also mediated by HS binding, demonstrated by ablating a positively-charged GAG binding region of E2 (K71A, K74A, K77A) [61]. More recent mechanistic analyses using CHIKV have shown GAG-dependent binding to be mostly mediated through E2 domain B while GAG-independent binding is mediated through domain A and that the extent of GAG dependence varies across CHIKV strains [110,111]. Discrete regions of E2 have also been implicated in viral persistence. Viral escape from phagocytic cell-mediated clearance and enhanced viremia and dissemination were found to be mediated by E2 K200R in CHIKV (Figure 5B) and ONNV and K251R in RRV, following the initial identification of K200R in a persistently circulating strain of CHIKV in immuno-compromised mice [62,63]. Mutational analysis revealed a lysine at these positions to be necessary for clearance [63].

While the mechanisms of E1 V72A and G313D were not elucidated, it is notable that residue 72 resides at the tip of domain II, which was later shown in SINV and other alphaviruses to function in the modulation of virulence. In a study of the evolution of CHIKV during natural transmission between Aedes aegypti mosquitoes and infant mice, the E1 variant V80I:A129V was identified in mosquito saliva and bodies and in mouse serum following transmission (Figure 5B) [51]. A recombinant virus with these mutations was generated and found to increase viral loads and lethality in mice. Mechanistic analyses revealed the mutations to increase fusion and particle stability in vitro. A subsequent study examined the mutational tolerance of E1 position 80, which is fully conserved among alphaviruses, and demonstrated that the amino acid at this position can modulate infectivity and dissemination [52]. While E1 variant V80L was attenuated in mice, double mutant V80L:V226A restored viral titers to wild-type levels, further demonstrating that discrete residues of E1 contribute to virulence. Additionally, SINV E1 V80L replication was shown to be attenuated in vitro, demonstrating the conserved functionality of this residue in another alphavirus. As discussed by Noval et al., residues 80 and 226 are both located at the tip of domain II of E1, and near a conserved glycerophospholipid binding pocket [52,112]. Their additional mechanistic analyses showing the role of residue 80 in cholesterol dependence together with previous reports that residue 226 is involved in cholesterol dependence in CHIKV, SFV, and SINV [54,55,56], support the functional importance of this region of E1 and possible implications in alphavirus virulence.

The significance of CHIKV E1 residue 226 (Figure 5B) was previously recognized following a 2005 outbreak when the virus’s increased ability to infect Aedes albopictus mosquitoes as compared to the primary vector Aedes aegypti was retrospectively attributed to the E1 mutation A226V, which gave rise to the Indian Ocean Lineage [53]. Following the emergence of the E1 A226V variant, a second-step adaptive mutation, E2 L210Q, was shown to also increase viral fitness in ae. Albopictus mosquitoes, although not to the extent of A226V, demonstrating the consequence in expanded circulation and epidemics brought on by the increased spread by ae. Albopictus. Interestingly, despite the high abundance of ae. Albopictus mosquitoes in South East Asia, the E1 A226V variant was not observed on the Asian lineage background, which was found to be due to an epistatic interaction between E1 residues 226A and 98T [113]. Further, two novel mutations E1 K211E and E2 V264A, found circulating in India and France, were shown to enhance infectivity in ae. Aegypti mosquitoes [114,115].

Also found to play a role in both vertebrate and mosquito infections is the glycosylation of the alphavirus glycoproteins (Figure 5B). RRV lacking N-linked glycosylation at E1 residue 141 (N141Q) led to increased clearance of the virus in mice and an associated increase in IFN-γ [57]. Loss of E2 glycan at residue 200 (N200Q) reduced infectivity in mosquitoes while altering the E2 262 glycosylated site (N262Q) had little effect [57]. Loss of SINV E1 glycosylation (N139Q or N245Q) also decreased virulence in mice, in addition to replication in mosquitoes [64]. The loss of SINV E2 glycosylation (N196Q or N318Q) actually increased virulence in mice and it was suggested to be due to increased heparan sulfate binding on mammalian cells [64]. The alphavirus glycoproteins have been implicated in critical roles throughout the viral lifecycle and as discussed here, evidence exists for various interactions with host components that contribute to virulence, but much of the underlying mechanism is not understood. Future studies will be valuable in further understanding how discrete residues, for example, are able to modulate alphavirus infection.

#### 2.2.3. E3

The E1-E2 heterodimer first exists as a p62-E1 intermediate before the cleavage of E3 from p62 (also known as pE2) to generate E2-E1. E3 is a small (~65 amino-acid) protein that functions in translocation of the structural polyprotein to the ER and in virus maturation via the cleavage of E3 from E2 [118,119]. During assembly, E3 associates with the glycoprotein spike complex to protect the fusion loop from low pH and prevent premature triggering [120]. While a direct role for E3 in virulence in animals has not been described, studies have described discrete residues of E3 involved in controlling the production of infectious particles in vitro. It was demonstrated that incorporation of uncleaved p62 can yield non-infectious particles and a single site mutation in SINV E3, C25R, restores infectivity [65]. Further mutational analysis of the conserved cysteine residues of alphavirus E3 proteins confirmed cysteine 25 and 19 of SINV E3 as critical for the production of infectious particles and provided a functional link for disulfide bond formation [121]. While additional studies are necessary to investigate other possible roles for E3, its function in regulating the processing and maturation of the glycoproteins implicates the small structural protein as potentially useful in the design of unique antiviral strategies.

#### 2.2.4. 6K and TF

Small accessory protein 6K and its minority frameshift product counterpart, TF, function in assembly and budding, and their specific roles in these processes have recently begun to come more to light, along with evidence for roles in virulence [20]. Mutations in 6K have been identified in epidemic strains of CHIKV, including the mutation L20M, which was detected during outbreaks in Mexico and Colombia [122,123]. Additional mutations of interest in the Colombian strains include 6K A47T/S, F48L, and A56V [123]. Although these mutations were sustained by epidemic variants, further study is required to address whether these mutations contribute to virulence. While 6K and TF are not required for the production of infectious particles, CHIKV, RRV, and VEEV mutants lacking the 6K gene and SINV mutants lacking TF or encoding altered versions of the protein are attenuated in mice [66,67,68,124]. It was suggested that decreased titers in the brain of VEEV Δ6K may indicate a role for the protein in neuroinvasiveness and/or crossing of the blood brain barrier [68]. Naturally arising deletions clustered near or spanning the 6K/E1 cleavage site and ribosomal frameshift site for TF translation in VEEV have also been described, further suggesting a significant role during infection [125]. A recent mechanistic study revealed SINV TF acts as an IFN-I antagonist in mice and primary macrophages and that palmitoylation of TF controls the capacity to antagonize IFN-I, establishing a novel mechanism responsible for TF-induced virulence that had not previously been identified [69]. Rogers et al. suggest palmitoylation may be necessary for TF interactions with host proteins or for proper localization to sites necessary for evading vRNA sensing, as they previously showed palmitoylation to be important in TF localization to the plasma membrane in addition to incorporation into particles, and particle morphology [69,126]. This group also used a domain-based mutational approach to characterize which regions of the protein control palmitoylation [70]. Cysteine residues of TF domain III (C35, C36, C38, and/or C39) were determined as the location of all palmitoylation, which occurs at a basal and maximal extent. Domain IV is crucial for the regulation of the ratio of basal and maximal palmitoylation and mutating cysteine residues there (C59, C62, and C65) results in only maximally palmitoylated TF [70]. Together with the activity of other viral proteins, TF-mediated antagonism of IFN-I production provides a multi-layered control over the host innate immune response, demonstrating the expansive multifunctionality of alphavirus proteins as determinants of virulence.

### 2.3. 5′ and 3′ Untranslated Regions

The alphavirus 5′UTR ranges in length from 27 to 85 nucleotides and contains functional sequence and structural elements important for replication, translation, and evasion of innate immune responses [127]. Early studies demonstrated that single point mutations in the 5′UTR cause attenuation in vivo, including at nucleotide 3 in VEEV, nucleotides 5 and 8 in SINV, and nucleotides 21, 35, and 42 in SFV [71,72,73,128]. In VEEV, the attenuation of G3A was attributed to increased IFN sensitivity [74]. Alphaviruses lack 2′-O methylation of the 5′ end of genomic RNA and have been shown to evade restriction by Ifit1, an IFN-stimulated gene with high affinity for unmethylated RNA [75,129]. Evasion of Ifit1 was shown to be dependent on nucleotide G3, which is able to alter Ifit1-RNA binding [75].

The alphavirus 3′UTR ranges from 87 to 723 nucleotides in length, contains the poly(A) tail, sequence and structural elements for replication, and binding sites of miRNAs and host proteins [127]. The 3′UTR of EEEV was predicted to contain four miR-142-3p, a hematopoietic cell-specific miRNA, binding sites spanning 260 nucleotides from position 11337 to 11596 responsible for restriction in murine myeloid cells [76]. Deletion of this region alleviated restriction of viral replication in these cells and altered infection in mice. Introducing three-point mutations to each of the miR-142-3p binding sites was sufficient to increase EEEV translation in murine myeloid cells in vitro. The authors suggest that 3′UTR-miRNA binding strategically limits viral replication in a cell-type specific manner, decreasing detection and ultimately leading to exacerbated disease [76]. The 3′UTR has also been implicated in host tropism and adaptation, as demonstrated by the deletion of nucleotides 31–293 in SINV that reduces replication in mosquito cells, but not in chicken cells [77]. This region contains a 19-nucleotide conserved element in which a single point mutation at position 7 renders the virus temperature sensitive in chicken cells while more dramatically attenuated in mosquito cells [77]. Deletion of this region in EEEV was observed following passaging in hamster cells further indicating that regions of the 3′UTR function in adaptation more in invertebrates more than vertebrates [130]. Naturally occurring lineage-specific patterns in the 3′UTR can be observed in alphaviruses, such as a 177-nucleotide duplication unique to CHIKV strains detected in the Caribbean islands and Mexico during a 2013/2014 outbreak [131,132]. As discussed extensively by Chen et al., such patterns that arise in the 3′UTR are likely due to a combination of adaptation to host and restriction in vectors [132]. Experimental passaging of a CHIKV mutant with a 258 nucleotide deletion in the 3′UTR demonstrated that passaging on a single host cell line increased viral fitness of the original deletion mutant and alternately passaged virus resulted in increased fitness on both hosts, with most changes occurring in the coding regions and demonstrating the impact of 3′UTR mutations on subsequent evolution and adaptation [133]. In a study that utilized immuno-deficient mice to assess the acquisition of adaptive mutations that facilitate persistent CHIKV infection in specific tissues, a 44-nucleotide deletion was detected in the 3′UTR from nucleotide position 11921 to 11964, along with a point mutation in E2 [62]. Mutational analysis showed that while the 3′UTR deletion alone was not sufficient to alter disease in mice, it contributed to enhanced effects in combination with the E2 mutation that enabled more rapid dissemination, further demonstrating the diverse functions of alphavirus UTRs [62]. With evidence for various roles in immune evasion, host tropism, dissemination, and persistence, the alphavirus UTRs are key contributors to the virulence of alphaviruses and further research is necessary to fully understand how these non-coding regions function in these processes.

## 3. Conclusions

Alphaviruses continue to pose an increasing threat to human health, creating the need for new preventative and therapeutic approaches. This review focuses on a number of discrete genomic regions and protein residues of multiple alphaviruses that have been shown to function in modulating virulence in vitro and in vivo. These molecular determinants of virulence represent subtle interactions between virus and host that are critical for understanding viral pathogenesis. Elucidating the mechanisms underlying these factors and the stability of mutations in the encoding genomic regions can greatly inform the development of new tools to combat these viruses. A rationally designed combination of stable targeted attenuating mutations based on these studies, for instance, can enhance the safety and success of live-attenuated vaccines, which are favorable in their ability to mimic natural infection and induce a robust immune response. Further, identifying conserved molecular determinants of virulence across alphaviruses is crucial for the development of pan-alphavirus antivirals and vaccine platforms. Pinpointing genomic regions important for virulence is also useful in the efficient characterization of new emerging viruses and viral variants. Paired with phylogenetics, epidemiological studies and clinical data, such functional information can be used to study of the evolution of virulence. The identification of additional determinants of altered immunogenicity, receptor binding, or vector tropism may be of key future interest to evaluate epidemic potential. Taken together, the virulence determinants reviewed here are only the tip of the iceberg of how alphaviruses cause disease. Future studies addressing how the alphavirus RNA genome and its encoded proteins contribute to virulence in vivo will be critical for our better understanding of the fundamental mechanisms of alphavirus biology.

## Figures and Tables

**Table 1:** Summary of alphavirus virulence determinants.

Gene/Region	Mutation/Element	Description	Reference
nsP1	I538T	Neurovirulence (SINV); IFN-I modulation (SINV, RRV)	[26,27]
nsP1	S79C, L224I	Musculoskeletal inflammation (RRV)	[28]
nsP2	SINV P726G, CHIKV P718S	IFN-I modulation; JAK-STAT inhibition	[29,30,31]
nsP2	A31T, N219T, S580L, Q619R	RRV IFN-I modulation; RIG-I, IRF3 expression	[32]
nsP2	_674_ATLG_677_	Transcription inhibition (CHIKV)	[33]
nsP2	KR649AA, P718S	UPR inhibition (SINV)	[34]
nsP3	T344, T345	Neurovirulence (SFV)	[35]
nsP3	SINV Opal-537, SFV Opal-469, CHIKV Opal-524	Pathogenesis in mice	[36,37,38]
nsP3	Residues 386–403	Neurovirulence (SINV)	[36]
nsP3	G32S/A and Y114A	Macrodomain activity, pathogenesis in mice (SINV)	[39]
nsP3	G3BP-binding motif 471–483; FXR-biding motif 531–547	Neurovirulence (EEEV)	[40]
nsP4	C483Y	Fidelity, fitness in mice/mosquitoes (CHIKV)	[41]
nsP4	G7R, E31G, S90T, and C482Y	Fidelity, fitness in mice/mosquitoes (VEEV)	[42]
nsP4	Undefined	EIF2α phosphorylation, UPR blocking (CHIKV, SINV)	[43]
Capsid	N-terminal NLS	Nuclearcytoplasmic transport blocking (VEEV)	[44]
Capsid	L48A, F50A	Nuclear import inhibition (VEEV)	[45]
Capsid	Residues 55–75	Host gene expression inhibition, IFN sensitivity, fitness in mice (EEEV)	[46]
Capsid	Undefined	PRR pathway inhibition (WEEV)	[47,48]
Capsid	K124A/K128A; K139A/K142A	RNAi blocking (SFV)	[49]
E1	V72A, G313D	Neurovirulence (SINV)	[50]
E1	V80I:A129V	Fitness in mice, stability (CHIKV)	[51]
E1	V80L	Infectivity, dissemination in mice/mosquitoes, cholesterol dependence (CHIKV)	[52]
E1	A226V	Vector tropism (CHIKV), cholesterol dependence (CHIKV, SFV, SINV)	[53,54,55,56]
E1	N141Q	E1 glycosylation, clearance and IFN-γ levels in mice (RRV)	[57]
E2	G55H, L209G, E70K	Neurovirulence, HS binding (SINV)	[50,58,59]
E2	R82G, E166K	GAG binding, disease severity, host response (CHIKV)	[58,59,60]
E2	K71A, K74A, K77A	Neurovirulence, GAG binding (EEEV)	[61]
E2	K200R (CHIKV, ONNV), K251R (RRV)	Fitness and cell-mediated clearance in mice	[62,63]
E2	N200Q	E2 glycosylation, mosquito infectivity (RRV)	[57]
E2	N196Q, N318Q	Fitness in mice, HS binding (SINV)	[64]
E3	C25R	PE2 processing (SINV)	[65]
6K	Undefined	Fitness in mice (CHIKV, RRV, VEEV)	[66,67,68]
TF	Undefined	IFN-I antagonism	[69]
TF	C35, C36, C38, C39	TF palmitoylation	[70]
5′UTR	Position 5 and 8 (SINV), 21, 35, and 42 (SFV)	Fitness in mice	[71,72,73]
5′UTR	G3A	IFN sensitivity, Ifit1 evasion (VEEV)	[74,75]
3′UTR	Position 11,337–11,596	miR-142-3p binding, immune detection (EEEV)	[76]
3′UTR	Position 31–293	Host adaptation, fitness in mosquitoes (SINV)	[77]
3′UTR	Position 11,921–11,964	Fitness in mice	[62]

## Data Availability

Not Applicable.
